# Menopause and Dermal White Adipose Tissue Depletion: Mechanistic Links, Adipogenesis, and Regenerative Therapeutic Replacement

**DOI:** 10.1111/jocd.70671

**Published:** 2026-01-11

**Authors:** Alan D. Widgerow, Mary E. Ziegler, Faiza Shafiq

**Affiliations:** ^1^ Center for Tissue Engineering University of California Irvine California USA; ^2^ Alastin, a Galderma Company Carlsbad California USA

**Keywords:** adipogenesis, adiponectin, dermal white adipose tissue, estrogen deficiency, extracellular matrix, magnolol, menopause, perilipin‐1, skin aging

## Abstract

**Background:**

Menopause is linked to typical skin changes such as textural alterations, loss of skin hydration, elasticity, thinning, and increased fragility. Dermal white adipose tissue (dWAT), a distinct fat component located in the dermis and involved in hair cycle regulation, antimicrobial peptide production, and extracellular matrix (ECM) modulation, decreases with aging and photodamage. Emerging evidence suggests that estrogen contributes to an inhibitory effect on dWAT and promotes fibrotic remodeling of adipose tissue of the dermis.

**Objective:**

To examine the mechanistic evidence linking menopause with the loss of dWAT and to suggest and highlight potential strategies for replacing dWAT with agents such as magnolol, which promote the conversion of pre‐adipocytes to adipocytes and restore lost fractions of the dWAT compartment.

**Methods:**

A review of the literature, a mechanistic examination, a histologic examination, and a clinical trial assessment were conducted.

**Results:**

The loss of dWAT and fibrotic replacement are likely a factor causally linked to the decrease in estrogen observed during menopause. Reduced dWAT produces fewer adipokines such as adiponectin, which is directly involved in skin health by promoting collagen and hyaluronic acid (HA) production. Evidence suggests that select non‐hormonal agents can offer a potential therapeutic benefit to menopausal skin promoting dWAT restoration through adipogenic pathways.

**Conclusion:**

dWAT depletion likely contributes to menopausal skin changes. Potential candidates for non‐hormonal alternatives to address these menopausal concerns are discussed in this paper.

## 1 Introduction

**FIGURE 1:**
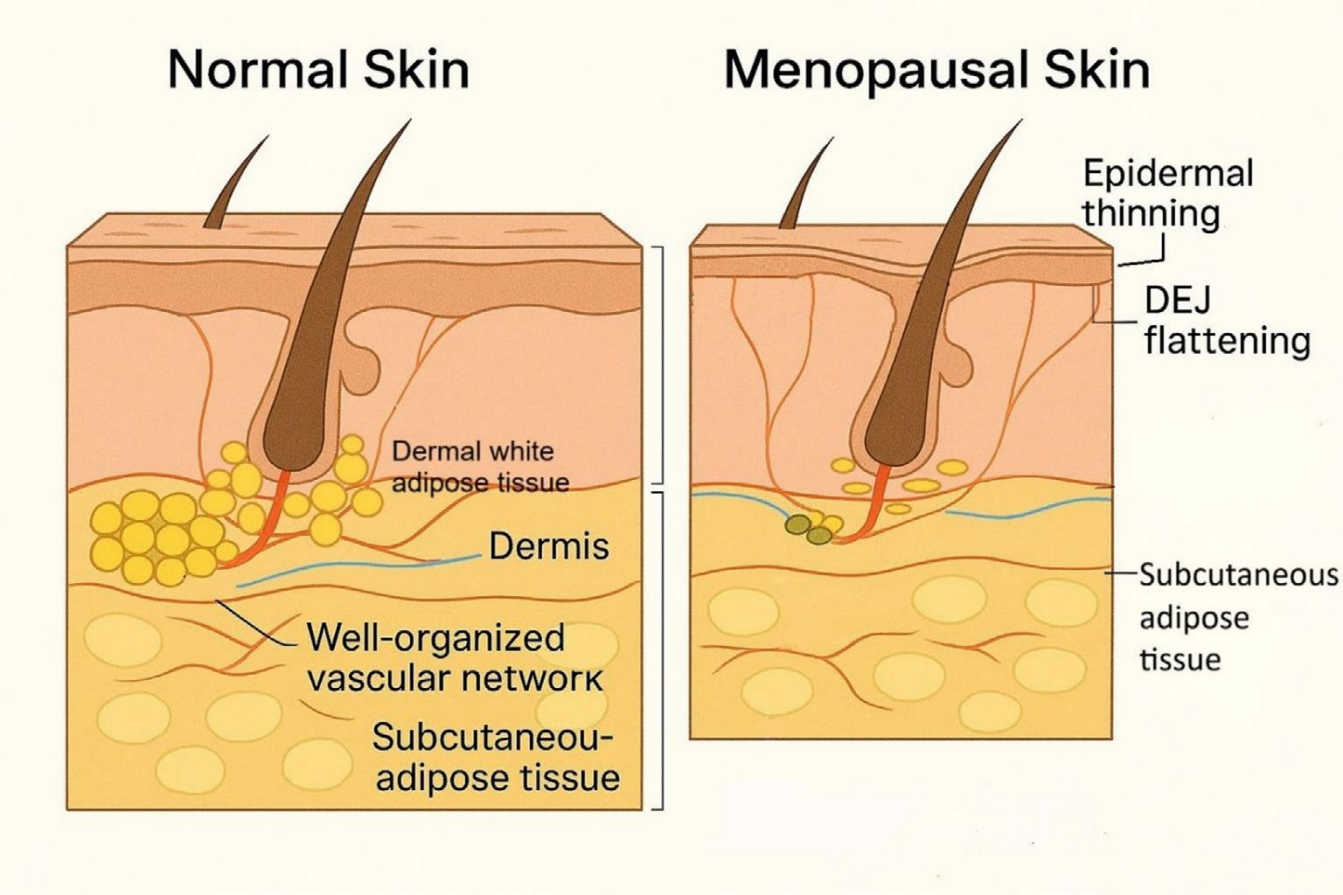
Comparative illustration showing the visible and histological changes that occur during menopause compared to normal skin, including epidermal thinning, dermo‐epidermal junction (DEJ) flattening, and loss of dWAT.

Menopausal skin exhibits visible changes, including thinning of the dermis, a rougher texture, increased laxity, and enhanced fragility. These changes begin during the perimenopausal stage, which is the transitional phase leading to menopause with further declines in estrogen levels. The perimenopausal years are characterized by an accelerated decline in skin quality, mainly due to decreasing estrogen levels, which impair collagen production and the integrity of the extracellular matrix (ECM) [1]. These declining estrogen levels and associated changes are linked to estrogen deficiency, which affects fat tissue by reducing essential fat deposits (Figure 1), decreasing important regenerative adipokines and active stem cell differentiation capacity, and increasing fibrosis in the dermal layer [2]. In addition, the dermo‐epidermal junction (DEJ) is recognized as a structural anchoring membrane fixing basal keratinocytes to the dermal papillary layer, providing a bridge between the dermis and epidermis, allowing cross signaling, supply of nutrients, and providing structural support for vessels and matrix [3]. Flattening and effacement of the DEJ with aging (and likely with menopause) from a healthy undulating appearance weakens the adhesive forces, decreases the distribution of shearing forces, and subjects the skin to injury and damage, with a predisposition to tears [4].

Within the dermis, dermal white adipose tissue (dWAT) is now recognized as a crucial regenerative compartment involved in hair cycle regulation, antimicrobial peptide production, and extracellular matrix (ECM) remodeling. As such, it acts as a hormonally responsive layer that supports skin health through paracrine signaling and interaction among multiple cell types and structural proteins [5, 6, 7]. Loss of dWAT disrupts this skin homeostasis and is increasingly acknowledged as a potential contributing factor to menopausal skin changes [7, 8].

### 1.1 dWAT: Biology and Function

dWAT is mainly found in the reticular dermis as small lobules of adipocytes, often located at the base of hair follicles. While dWAT is often depicted as a continuous layer of intradermal fat [9], this understanding largely stems from rodent studies. In human skin—especially on the face—we believe that dermal fat usually appears as discontinuous, perifollicular clusters and scattered adipocyte progenitors within the reticular dermis. These progenitors can look similar to fibroblasts in routine histology, which may lead to them being overlooked. Therefore, we describe human facial dWAT as a functional dermal fat compartment that is structurally diverse rather than a uniform layer.

These fat cells are encased by collagen bundles and blood vessels, together creating a regenerative niche that has only recently been recognized. Under the influence of paracrine signals or specific active agents, these pre‐adipocytes can develop into adipocytes with regenerative capabilities. Conversely, factors like ultraviolet (UV) injury have been known to cause pre‐adipocytes to transform into fibroblasts or myofibroblasts, leading to a fibrotic environment rather than a regenerative one [8, 10, 11, 12]. A similar mechanism can be hypothesized for the menopausal skin environment as well.

dWAT is involved in:

- Hair cycle regulation—dWAT expands during anagen and shrinks during catagen, supporting stem cells, lipids, and adipokines.
- ECM modulation—adipokines, especially adiponectin, directly impact fibroblasts by stimulating collagen, elastin, and HA production. dWAT also enlarges in the early stages of wound healing.
- Immune regulation—dWAT adipocytes produce cathelicidins, antimicrobial peptides, that influence local inflammation and infection. This is only during reactive adipogenesis, under infectious/inflammatory cues.
- Mechanical buffering—dWAT offers cushioning for the dermis and acts as a source of nutritional and metabolic support.

UV light and aging can affect dWAT volume but these changes are dependent upon UV exposure timing and intensity. Decreasing dWAT volume can lead to impaired pre‐adipocyte to adipocyte differentiation, altering stem cell capacity, and promoting preadipocyte to fibroblast/myofibroblast transition, which leads to dermal fibrosis, skin atrophy, fine lines, and loss of elasticity [10, 12].

### 1.2 Menopause and Reduced dWAT

Estrogen deficiency during menopause does not lead to significant changes in estrogen receptor (ER) expression in the dermis. Immunohistochemical analysis shows no difference in ERα and ERβ expression between male and female human skin. However, ERβ expression is notably decreased in the epidermis of individuals aged 70 or older [13]. Thus, the impact of estrogen on aging human skin has been clarified by comparing post‐menopausal women receiving estrogen replacement therapy with those who are not. Oral estrogen treatment is linked to increased epidermal thickness in female human skin, along with larger keratinocyte volume and more defined rete ridges [14]. It is important to differentiate facial skin changes with menopause from other anatomic areas. In facial skin and its associated dWAT, estrogen receptors (alpha and beta) are found in dermal fibroblasts, hair follicles, glands and adipose pre‐adipocyte and adipocytes. Lower estrogen signaling is linked to decreased ECM integrity with thinning and lack of support for fat tissue resulting in atrophy and downward displacement of the fat. Although dWAT has not been examined and mapped as thoroughly as larger fat deposits, it is likely that estrogens help maintain subcutaneous facial fat as well as superficial dermal fat deposits and its loss reduces the capacity for overall fat formation, while also impacting skin structure and dermal fibroblast function [15].

In contrast, in abdominal and visceral adipose tissue, menopause consistently causes a redistribution of fat toward visceral white adipose tissue (vWAT), leading to metabolic problems. Declining estrogen alters how fat is metabolized and how receptors are expressed in these areas, decreasing the likelihood of healthy subcutaneous fat growth and increasing visceral fat cell enlargement, inflammation, and scarring [16, 17, 18]. During menopause, reduced estrogen levels suppress adipogenesis and adipocyte distribution likely leading to decreased local adipokine levels like adiponectin and increased superficial fibrosis. Histologically, this manifests as reduced dWAT, a loss of perilipin‐1 (PLIN1) positive adipocytes, and fibrotic replacement [19, 20, 21]. However, the relationship between estrogen status and adiponectin is inconsistent across studies. Some work reports no significant change in circulating adiponectin with menopause, while others show increases or decreases. Importantly, almost all of these data refer to systemic (blood) adiponectin levels which is important because local adipokine expression may differ from circulating levels, and facial adipose depots have depot‐specific biology not routinely sampled in human studies [22]. We hypothesize that non‐hormone‐based agents can be used to reactivate dWAT thereby improving topical skin changes that occur during menopause.

### 1.3 Magnolol and Adipogenesis

To identify an active agent that promotes pre‐adipocyte to adipocyte conversion and thereby replenishes dWAT tissue, a series of in vitro studies was conducted. Primary human adipocyte cultures treated with magnolol showed phenotypic changes, including lipid droplet deposition, upregulation of the PLIN1, FABP4 (fatty acid binding protein 4), and ADIPOQ (adiponectin) genes, and a significant increase in adiponectin protein secretion [23]. The bioactive compound magnolol was sought as a potential candidate for activating adipogenesis via the PPARγ and CEBPα pathways. PPARγ serves as the dominant effector of adipogenesis [23, 24]. This mimics estrogenic adipogenic signaling and supports magnolol's effect in stimulating dWAT.

### 1.4 PLLA (Poly‐l‐Lactic Acid) SCA (Sculptra Collagen Activator)—PLLA‐SCA and Adipogenesis

Healthy new fat tissue is increasingly recognized as a key regenerative organ [25]. Recent studies have uncovered a new potential pathway involving PLLA‐SCA. A bulk RNA‐seq analysis assessing skin biopsies after PLLA‐SCA injection demonstrated the upregulation of genes related to adipogenesis pathways (FABP4, ADIPOQ, CIDEC, LIPE), indicating an adipocyte regenerative potential [26]. Additionally, PLLA‐SCA influences macrophage polarization, encouraging M1 to M2 conversion, a necessary step in turning off inflammation and promoting regeneration. This process is mediated through IL‐6, IL‐10, and IL‐4 pathways [26].

Clinically, PLLA‐SCA enhances dermal thickness, elasticity, and texture, going beyond the initial biostimulatory neocollagenesis thought to be its mechanism of action. The addition of regenerative adipogenic components and ECM modulation firmly places this technology in the ‘regenerative filler’ category [26, 27, 28]. When combined with the RSC 2.0/magnolol topical therapy, we can propose a hypothetical clinical framework program that can offer a dual adipogenic approach that targets both dermal and subdermal loss characteristic of menopause (Table 1).

**TABLE 1:** Proposed clinical framework.

Phase	Timeframe	Intervention	Mechanism
1. Baseline prep	Weeks 0–2	RSC 2.0 twice daily	Preadipocyte activation, PLIN1/FABP4 upregulation
2. Deep adipogenic booster	Week 0–4	PLLA‐SCA injection (8 mL dilution)	FABP4/PLIN1/CIDEC activation, macrophage modulation
3. Remodeling	Weeks 4–12	Continue RSC 2.0 ± energy device	Adiponectin‐mediated ECM deposition and collagen remodeling
4. Maintenance	> 12 weeks	Ongoing RSC 2.0, annual PLLA booster	Sustained adipogenesis and dermal renewal

## 2 Summary and Conclusion

The decrease in estrogen linked to menopause disrupts adipogenic pathways and signaling, reducing dWAT and affecting dermal thickness and structure. Magnolol promotes pre‐adipocyte differentiation, and PLLA‐SCA encourages adipogenesis, helping to restore healthy new adipose tissue and modify the ECM environment. Clinical evidence of improved plumpness, texture, and elasticity, along with histologic evidence of neoadipogenesis, supports this biological strategy. This dual approach to menopausal skin, involving both the injectable booster and topical maintenance, introduces a new therapeutic paradigm focused on—adipogenic rejuvenation for menopausal skin. Although direct human measurement or quantification of dWAT in menopause remains to be established, current evidence strongly suggests the plausibility of dWAT interventions.

## Ethics Statement

Ethical guidelines for Wiley were followed.

## Consent

Informed consent was obtained in all studies referred to in this publication.

## Conflicts of Interest

Dr. Alan D. Widgerow is Chief Scientific Officer of Galderma. Dr. Faiza Shafiq is Director Clinical Research Alastin Skincare Inc., a Galderma company. Dr. Mary E. Ziegler is a consultant to Alastin Skincare Inc., a Galderma company. This paper reports the suspected link of dWAT and menopause.

## Data Availability

The data that support the findings of this study are available from the corresponding author upon reasonable request.
